# BITS2019: the sixteenth annual meeting of the Italian society of bioinformatics

**DOI:** 10.1186/s12859-020-03708-x

**Published:** 2020-09-16

**Authors:** Alfonso Urso, Antonino Fiannaca, Massimo La Rosa, Laura La Paglia, Giosue’ Lo Bosco, Riccardo Rizzo

**Affiliations:** 1 ICAR-CNR, Institute for high performance computing and networking, National Research Council of Italy, Palermo, 90146 Italy; 2 Department of Mathematics and Computer Science, University of Palermo, Palermo, 90128 Italy

## Abstract

The 16th Annual Meeting of the Bioinformatics Italian Society was held in Palermo, Italy, on June 26-28, 2019. More than 80 scientific contributions were presented, including 4 keynote lectures, 31 oral communications and 49 posters. Also, three workshops were organised before and during the meeting. Full papers from some of the works presented in Palermo were submitted for this Supplement of BMC Bioinformatics. Here, we provide an overview of meeting aims and scope. We also shortly introduce selected papers that have been accepted for publication in this Supplement, for a complete presentation of the outcomes of the meeting.

## BITS meetings

Meetings of the Bioinformatics Italian Society (BITS) [1] are a series of international conferences held annually in Italy since 2004 that highlight keynote talks by excellent scientists in bioinformatics and its applications, oral presentations of state-of-the-art research in bioinformatics and computational biology, and poster sessions on the latest research progress.

## BITS 2019

## Scope and rationale

The event BITS 2019: 16th Annual Meeting of the Bioinformatics Italian Society (BITS) was held on June 26-28 2019, in Palermo, Italy.

The goals of the BITS association are the study, development and spreading of Bioinformatics in a scientific, academic, technological and industrial environment. The BITS meetings highlight keynote talks by excellent scientists in bioinformatics and its applications, presentations of state-of-the-art research in computational biology, and poster sessions on the latest research progress. Many aspects of the bioinformatics disciplines have been covered during the event such as Algorithms in Bioinformatics, Storage, Analysis and Visualisation of Biological Databases, Biological Image Analysis, Computational RNA biology, Epigenetics and Epigenomics, Genome Organization and Gene Regulation, Machine Learning in Bioinformatics, Methods for Single-cell Analysis, (Multi-)Omics Data Integration and Analysis, Metagenomics, Molecular Dynamics Simulations, Molecular Evolution, Pharmacogenomics, Proteomics, Structural Bioinformatics, Systems Biology and Interaction Networks, Translational Bioinformatics, Education and Training, Industry Tracks. The event gave to participants the opportunity of introducing and discussing new methods, theoretical approaches, algorithms, tools, and platforms, as from tradition of BITS previous events. For a full list of topics, see the meeting web site [2].

## Scientific programme

The programme included 4 invited keynote lectures, 31 oral presentations and 49 posters. Keynote speakers included Emmanuel Barillot, Silvio Bicciato, Raffaele Giancarlo and Roded Sharan. As BITS meetings tradition, local chairs organised a special session in order to emphasise a hot topic in bioinformatics research. This years’ special session was entitled: “Bioinformatics challenge in precision medicine”.

### Keynote speakers

Emmanuel Barillot is the head of the Department of Epidemiology, Biostatistics, Bioinformatics and Computational Systems Biology of Cancer, a joint Institut Curie and INSERM research department, in partnership with Mines ParisTech. His research interests are in the field of computational systems biology of cancer, with a focus on tumorigenesis and tumour progression. Computational approaches include original statistical methods and biological network modelling approaches considering high-throughput heterogeneous biological data and their integration. In his talk “Some new challenges in computational molecular oncology”, Dr Barillot presented the most recent strategies for the analysis of the molecular profiles of tumours in order to support precision medicine in oncology. In particular, the talk focused on single-cell analysis with case studies on medulloblastoma, paediatric cancer, and single-cell trajectory inference.

Silvio Bicciato is full professor in Industrial Bioengineering at the University of Modena and Reggio Emilia. In the last decade, he and his research group designed and developed computational methods, algorithms, and tools for the multi-dimensional integrative analysis of genomics, transcriptomics, epigenomics, proteomics, and phenotypic data in the field of onco-genomics, immunogenomics, and neurosciences. His current research activities are addressed to the development of computational methods to resolve the spatial, architectural and social heterogeneity of cells within a tissue, to capture the evolution of cellular ecosystems in time, and to detect the dynamics of epigenetic regulation. Prof Bicciato presented a talk with the title “Integrative bioinformatics for precision medicine”. In his talk, it is shown how the paradigm of heterogeneous data integration is essential in bioinformatics as it allows to translate in silico results in clinical practice that impacts on human health and well-being. In particular, Prof. Bicciato spoke about computational strategies and new bioinformatics challenges in order to collect molecular signals and to train mechanistic and predictive models.

Raffaele Giancarlo is full professor of Computer Science in the Department of Mathematics and Computer Science at the University of Palermo. His research interests are Algorithms and data structures, Classification and Machine Learning, Computational Biology and Bioinformatics, Data compression, Information retrieval. His recent research activities have concerned the study on the organisation and dynamics of chromatin by Theoretical Computer Science approaches such as Combinatorial and Informational Methodologies. Prof. Giancarlo presented the talk “DNA combinatorial messages and Epigenomics: The case of chromatin organisation and nucleosome occupancy in eukaryotic genomes”. In his speech, he focused on the importance of epigenomic studies for understanding the working mechanism of cells in Eukaryotic organisms. The study of chromatin, a fibre composed of a DNA-protein complex, is of particular importance because its organisation and assembly influence some biological mechanism such as gene expression regulation and DNA repair. In that talk, combinatorial and informational methodologies were provided in order to understand better the role of chromatin, such as packaging and nucleosome positioning.

Roded Sharan is full Professor in the School of Computer Science, Tel Aviv University. As head of its research group, his current research interests are about the analysis of biological networks and their applications to medicine. In his lecture “Harnessing protein networks to elucidate disease mechanisms”, Prof. Sharan described how integrating several molecular measurements into a single computational framework can improve the understanding of protein networks and diseases. The talk illustrated that network modelling and analysis are, in fact, among the most suitable strategies for correlating novel genes and modules with diseases and discovering this way new potential therapeutic targets.

### Special session

2019 edition of BITS conference proposed a special session named “Bioinformatics challenge in precision medicine”. Precision, or personalised, medicine is a hot topic in current bioinformatics and biomedical research. The growing amount of multi-omics data such as genomics, transcriptomics, proteomics data, is promoting a considerable shift in the study of biomedical sciences, defining new methods and approaches for diagnostics and therapeutics of human diseases. These models flow into the paradigm of precision medicine, whose goal is to provide the best available care for each individual, requires that researchers and healthcare providers have access to large sets of health and disease-related data linked to individual patients. In this context, collection and analysis of these -omics data, as well as their integration at different scales with other biomedical data, such as bioimages, clinical and lifestyle data, represent the first challenge in precision medicine studies. Tools, algorithms and protocols for data management, including privacy and security issues, should lead to the adoption of platforms and services in healthcare, focusing on new clinical treatments designed to target a single patient.

That special session was devoted to collect contributions on every aspect of precision medicine trending topics, including -omics data integration, data management, data analysis techniques, and their employment in development of new tools and services with applications for healthcare.

BITS 2019 special session was composed of four oral presentations and one keynote speech by Silvio Bicciato.

### Workshops

The first workshop was a satellite event held on June 25th 2019 at the Cefalù Municipal theatre. The event was organised by the Institute of Molecular Bioimaging and Physiology of the National Research Council of Italy (IBFM-CNR), and the title of the workshop was “Applications of computer science in diagnostic imaging”. Computer science techniques represent a capable help to the clinician in reducing the bias linked to the intrinsic subjectivity of human evaluation. Today, computer-assisted approaches allow providing the clinician with tools capable of supporting him, of reducing the operator’s dependence and, consequently, to improve the care and follow-up process. The aim of the workshop was to show how information technology can support the clinicians work, providing them with advanced tools able to assist them in the reporting and decision-making processes in typical and routine tasks, such as segmentation, identification and of regions or volumes of interest, the quantification and analysis of medical images. The workshop presentations highlighted a Radiomics perspective focusing on extracting and mining a large number of medical imaging features. The workshop outcomes that Radiomics can be a valid approach to improve individualised treatment selection and monitoring.

The second workshop titled “Algorithms and Tools for the Analysis of Big Omics Data” was held on June 26th, the morning before the Bits 2019 event. It was organised by Fabio Fassetti (DIMES,Università della Calabria), Giosué Lo Bosco (DMI, Università degli Studi di Palermo), Cinzia Pizzi (DEI, Università degli Studi di Padova) and Simona E. Rombo (DMI, Università degli Studi di Palermo). The goal of the workshop was to provide to participants the opportunity of introducing and discussing new methods, theoretical approaches, algorithms, tools, and platforms that are relevant to the bioinformatics community for the extraction, integration and analysis of big omics data. Two invited speakers discussed big data challenges in cancer and single-cell genomics. In particular Fabio Vandin from the University of Padua, Italy, during his talk titled “Finding Patterns in Cancer Genomes: Challenges and Algorithms” talked about his recent works on the development of algorithms to identify reliable and significant patterns from measurements of a large collection of tumours. The second invited was Luca Pinello from the Massachusetts General Hospital Research Institute and Harvard Medical School, USA, who presented in the talk titled “Computational Methods for Single-Cell Genomics and Genome Editing” two recent contributions by his lab about single-cell analysis and genome editing. The workshop also hosted eight oral presentations, related to abstracts which were submitted and reviewed by the organising committee.

The last workshop was the Young BITS event, that was held in order to offer an overview on Research funding opportunities for young scientists, as it is one of the main challenges to face at the beginning of a scientific career. The workshop, organised by RSG-Italy and youngBITS, aimed to share experiences of young researchers about all aspects of participating and obtaining national, European and international projects. To this purpose, tree of the speakers of the congress, participated to the tutorial to tale their own experience to young research audience: Simona Rombo from the University of Palermo, Marco Necci from University of Udine and Luca Pinello from Harvard Medical School. After speakers’ presentations, there was a constructive and critical debate in which different aspects of financial opportunities and strategies were discussed with all the audience.

## Selected papers

The Guest Editors of the Supplement selected an Editorial Committee by paying attention that topics of submitted manuscripts were adequately covered. The Guest Editors managed the reviewing process for each manuscript, which was assigned to one of them according to his/her expertise. Two or more referees were selected for each submission: 25 referees were involved overall. Authors were invited to submit an improved version of their manuscript after the reviews were collected. The Guest Editors made the final recommendation for each manuscript and, at the end of this process, ten papers were accepted.

### Brief description of selected papers

The analysis of sequenced metagenomes from various environmental samples is an open challenge in translational bioinformatics [3]. The paper “Metagenomic analysis through the extended Burrows-Wheeler transform” [4] providing a tool for Taxonomic classification of metagenomic reads called LiME (Lightweight Metagenomics via eBWT). LiME is a combinatoric approach, based on the extended Burrows-Wheeler Transform (eBWT) enhanced with other data structures such as the document array (DA) and the longest common prefix (LCP) array. LiME belongs to the class of alignment-free, mapping-free and assembly-free methods for comparing sequences, with an internal memory usage depending only on the number of reads that one wants to examine at the same time. Experiments have been carried out on NGS data from two simulated metagenomes and a real one from the Human Microbiome Project. Experiments show that LiME is competitive on the simulated data with other commonly used taxonomic classifiers, such as Magic-BLAST [5], CLARK-S [6], Centrifuge [7], Kraken 2 [8] and TaxMaps [9]. On the real metagenome, LiME is comparable to MagicBlast [5].

In the genomic era, second and third next-generation sequencing (NGS) technologies strongly changed the biological approach to the study of life sciences. Accordingly, the explosive growth of sequencing data also increased the development of efficient data analysis tools for facing different challenges. A considerable effort was addressed in finding genomic variations linked to human disease [10]. The paper “Variable-Order Reference-Free Variant Discovery with the Burrows-Wheeler Transform”, by Prezza et al. [11], presents ebwt2InDel, a new tool for the variant calling process of a genome(s) or exome(s) sequence. This tool efficiently deals with the identification of variants from NGS data, in order to characterise individual, trait or population. The proposed reference-free tool for the detection of single nucleotide polymorphisms (SNPs), insertions and deletions (INDELs) is based on an extended version of the Burrows-Wheeler Transform (eBWT) [12]. It represents an improvement of the previous works by the same authors [13] where they proposed an alignment-free framework for the detection of SNPs based on BWT. In this work, the authors also provided a parallel version of the ebwt2InDel tool, that introduce the clear advantage of reducing the computational cost. However, it is limited to discover only SNP variants. In order to test the performance of the proposed tool, authors tested their algorithm (both the sequential and the parallel version) on synthetic and real Human chromosome 1 NGS data, and on a real whole-genome sequencing experiment.

The paper “A computational framework for modelling and studying pertussis epidemiology and vaccination” [14] presents a framework for the study of epidemiological systems. The authors pointed out that the construction of a model in a challenging task so that they focused the attention on guide the user and to improve portability and integration to existing frameworks. The tool uses the Petri Nets formalism [15] to help the user to simplify the model creation phase and the Docker framework in order to allow easy installation and use of the tool. The paper presents a case study of the framework by investigating the pertussis epidemiology in Italy.

In the research area of proteomics, Savojardo et al. presented the work “Large-scale prediction and analysis of protein submitochondrial localisation with DeepMito” [16]. In that work, authors applied their previously developed DeepMito algorithm [17] on large scale data. They performed proteome-wide prediction of submitochondrial localisation on representative proteomes of five species including human, mouse, yeast, fly and Arabidopsis thaliana. Moreover, they functionally annotated protein data using gene ontology (GO) annotations found in UniprotKB [18] and when necessary predicted with Bologna Annotation Resource (BAR) tool [19]. The resulting 4307 annotated mitochondrial proteins were collected in a novel database called DeepMitoDB. DeepMitoDB was compared against other similar databases such as IMPI [20], MitoCarta [21] and the Human Protein Atlas – Subcellular Localization (HPA-SL) [22]. Finally, the authors presented a practical use case of DeepMitoDB, based on the analysis of the human Nocturnin protein.

The goal of the procedure developed in radiomics field is to construct a predictive model, for example, for patient outcomes, through features automatically extracted from medical images. The first step of all these procedures is the automatic identification of a Biological Target Volume (BTV) because the manual procedure has high variability and gives not reproducible results. In the paper “A preliminary PET radiomics study of brain metastases using a fully automatic segmentation method” [23] the authors developed a system to discriminate between patients with brain metastases able to respond to the treatment or not, based on an already studied fully automatic segmentation procedure for PET images [24, 25]. The paper uses the CGITA toolbox [26] for automatic extraction of more than one hundred features from the image of brain metastases and presents an original statistical method to define the minimum set of features useful to discriminate between responder or not responder patients.

The study of microRNAs (miRNAs) is of great importance to the understanding of relevant mechanisms related to various diseases. They behave as competitive endogenous RNA (ceRNA), acting as natural miRNA sponges to inhibit miRNA functions and modulate the expression of RNA messenger (mRNA). Understanding the ceRNA–miRNA–mRNA crosstalk is of fundamental importance to increase the functional information across the transcriptome. The work “miRTissue_*ce*: extending miRTissue web service with the analysis of ceRNA-ceRNA interactions” [27] presents a web service to search for ceRNA interactions in several cancer tissue types. It is an improvement of the miRTissue [28], a web service that allows searching for a tissue-specific characterisation of miRNA-target interactions in human. miRTissue_*ce* implements a computation pipeline based on the state-of-the-art algorithm for inferring ceRNA-ceRNA interactions, and it provides an easy way to search for ceRNA interactions in several cancer tissue types. These features characterise miRTissue_*ce* with respect to other web services available for similar purposes. The adoption of miRTissue_*ce* has been described for the case of important Bioinformatics scenarios regarding cancer study such as therapeutics analysis, biomarker discovery and use of interaction networks.

Virtual Screening (VS) is a computational technique applied for drug design. In VS, the complexity of the algorithms used behind the screening leads to generate models with different prediction reliability. The optimal design of the compound is a multidimensional issue involving different aspects of Chemistry and Biology, which can be faced using Machine Learning (ML) techniques due to their possibility to access and mining large data sets containing heterogeneous information. In the last few years, Deep Learning Techniques, and particularly Convolutional Neural Networks (CNN) gained more and more impact on drug design and VS due to the enormous increase of the prediction accuracy in any stages of this process [29]. Deep Neural Networks (DNN) have been used for predicting different properties demonstrating reliable and robust prediction capabilities with high sensitivity when used on different targets [30, 31]. The paper “Convolutional Architectures for Virtual Screening” [32] by Mendolia et al., proposes a novel CNN architecture trained on the molecular fingerprints to predict the biological activity of candidate medical compounds versus the CDK1 protein target, using their IC50 value. The proposed approach uses molecular fingerprints as the embedding for a VS deep neural architecture both alone and combined as bi-dimensional binary matrices. Moreover, authors propose the design of several architectures for achieving good performances in both early screening and a mature stage, when precise discrimination between active and inactive molecules is needed.

In epigenomics studies, the structure and functionalities of nucleosomes are of fundamental importance. Nucleosomes are the fundamental unit for chromatin packaging [33], and they are involved in many biological events such as gene regulation, replication and recombination [34]. The paper “CORENup: A Combination of Convolutional and Recurrent Deep Neural Networks for Nucleosome Positioning Identification” by Amato et al. [35], present a deep learning approach for detecting nucleosome positioning considering only DNA sequences. CORENup is an extension of authors’ previous works [36, 37] and it presents a deep neural network model composed of a convolutional neural network (CNN) and a recurrent neural network (RNN) that work in a parallel manner. This way, the model can capture either non-periodic and periodic DNA features. CORENup has been trained and tested, considering several datasets belonging to different biological species. Classification results, in terms of many statistical scores, have been compared against the performance of current state-of-the-art tool: LeNup [38]. CORENup model demonstrated to outperform the LeNup algorithm almost in all the cases, with improved training time.

As is well-known MicroRNA (miRNAs) act as post-transcriptional regulation molecules regulating the expression level of messenger RNAs (mRNAs). For this reason, miRNAs are critical in many biological processes, and they play an essential role as biological markers for many diseases. Because a miRNA can bind many mRNAs and several miRNAs can bound a mRNA, a crucial issue is the ability to predict the targets of the endogenous miRNAs, in order to understand the processes they are involved. Moreover, miRNAs regulation activity depends on the recognition of binding sites located on mRNA molecules. MicroRNA target prediction algorithms are generally based on Watson-Crick base-pair matching [39, 40]. Other methods use the miRNA expression profile as additional information [41, 42], or the free energy between the binding sites [43, 44]. In this research area Bertolazzi et al. present the paper “An improvement of ComiR algorithm for microRNA target prediction by exploiting coding region sequences of mRNAs” [45]. The paper is an upgrade of ComiR (Combinatorial miRNA targeting) [46], an innovative algorithm to predict targets of endogenous miRNAs that incorporates miRNA expression in a thermodynamic binding model and associating each gene with the probability of being a target of a set of miRNAs. The new algorithm introduces information about the binding sites contained in the coding region of the genes. Authors show that the information contained in the coding region significantly improves the accuracy of ComiR predictions.

The research on protein-protein interactions and protein structures is rapidly increasing. Indeed, a variety of experimental and computational techniques can be used to identify possible protein binding partners of a given protein [47]. In this context, the study of protein-protein docking models is a challenging task, as experimental determination of protein-protein complexes are quite difficult compared with the determination of the isolated components because one protein can interact with a multitude of different partners. Thus we need to obtain accurate models of protein-protein complexes, and also adequate and proper scoring protein-protein docking models. This last task is also an object of assessment in CAPRI (Critical Assessment of Predicted Interactions), a community-wide blind docking experiment [48]. Oliva et al., in the paper entitled “The CASP13-CAPRI Targets as Case Studies to illustrate a Novel Scoring Pipeline Integrating CONSRANK with Clustering and Interface Analyses [49], propose a pipeline combining a consensus method called CONSRANK (CONSensus RANKing) and an algorithm called Clust CONSRANK. The former ranks docking models based on their ability to match the most conserved (or frequent) inter-residue contacts in the ensemble they belong to; the latter introduces a contact-based clustering of the models as a preliminary step of the CONSRANK scoring process. In CAPRI assessment authors pipeline for scoring protein-protein docking models achieved the first position in the Top-1 and Top-10 Scorers’ Rankings and in the Prediction of Binding Interface of the CASP13- CAPRI46 Experiment. Probably, the highly successful performance obtained in CAPRI Experiment was obtained through the introduction of some flexibility in the final model selection and ranking, and through differentiating the adopted scoring approach depending on the targets under investigation. The method proposed by the authors has great potential in many applications implying the use of structural information for protein-protein complexes, including drug discovery, and in rational drug design targeting protein-protein interactions.

## Data Availability

Not applicable

## Abbreviations

BITS: Bioinformatics Italian Society
INSERM: Institut National de la Santéet de la Recherche Médicale
CNN: Convolutional Neural Network
RNN: Recurrent Neural Network
NGS: Next-generation sequencing
SNP: Single nucleotide polymorphism
INDEL: Insertion or deletion
BWT: Burrows-Wheeler transform
LeNup: Learning Nucleosome positioning
BAR: Bologna Annotation Resource
HPA-SL: Human Protein Atlas –Subcellular Localization
IBFM-CNR: Institute of Molecular Bioimaging and Physiology of the National Research Council of Italy
CAPRI: Critical Assessment of Predicted Interactions
